# Steric interactions lead to collective tilting motion in the ribosome during mRNA–tRNA translocation

**DOI:** 10.1038/ncomms10586

**Published:** 2016-02-03

**Authors:** Kien Nguyen, Paul C. Whitford

**Affiliations:** 1 Department of Physics, Northeastern University, Dana Research Center 111, 360 Huntington Avenue, Boston, Massachusetts 02115, USA

## Abstract

Translocation of mRNA and tRNA through the ribosome is associated with large-scale rearrangements of the head domain in the 30S ribosomal subunit. To elucidate the relationship between 30S head dynamics and mRNA–tRNA displacement, we apply molecular dynamics simulations using an all-atom structure-based model. Here we provide a statistical analysis of 250 spontaneous transitions between the A/P–P/E and P/P–E/E ensembles. Consistent with structural studies, the ribosome samples a chimeric ap/P–pe/E intermediate, where the 30S head is rotated ∼18°. It then transiently populates a previously unreported intermediate ensemble, which is characterized by a ∼10° tilt of the head. To identify the origins of head tilting, we analyse 781 additional simulations in which specific steric features are perturbed. These calculations show that head tilting may be attributed to specific steric interactions between tRNA and the 30S subunit (PE loop and protein S13). Taken together, this study demonstrates how molecular structure can give rise to large-scale collective rearrangements.

## Abstract (web summary)

During protein elongation, the translocation of mRNA and tRNA molecules across the 30S ribosomal subunit is associated with large-scale motions of the 30S head domain. Here the authors carry out MD simulations to probe the associated steric interactions and identify novel tilting motions during the late stages of translocation.

## Graphical abstract

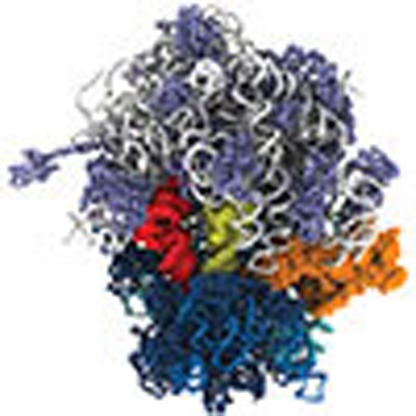

Ribosomes are complex multicomponent molecular machines that are responsible for the synthesis of proteins in all organisms^1^,^2^,^3^,^4^,^5^,^6^. In bacteria, the 70S ribosome (2.4 MDa) is composed of two subunits: a ‘large' 50S subunit and a ‘small' 30S subunit (Fig. 1a). The 50S subunit consists of two ribosomal RNA (rRNA) molecules (23S and 5S) and over 30 proteins. The 30S subunit contains one rRNA molecule (16S) and roughly 20 proteins. Both subunits have three binding sites (A, P and E) to which transfer RNA (tRNA) molecules sequentially bind during protein synthesis. Following accommodation of an aminoacyl-tRNA (aa-tRNA) molecule into the A site, the nascent peptide chain is transferred from the P-site tRNA to the aa-tRNA in the A site, thereby extending the peptide chain by one amino acid. Subsequently, the A- and P-site tRNAs move (along with the associated mRNA) by one binding site, into the P and E sites. The displacement of tRNAs between binding sites is called translocation, and it results in a vacant A site. By vacating the A site, the next mRNA codon may be read by another incoming aa-tRNA molecule.

The process of translocation is generally described in terms of two steps: (1) tRNA hybrid-state formation and (2) translocation of the mRNA–tRNA codon-anticodon pair^1^,^4^,^7^,^8^. During the first step, the acceptor ends of the A and P site tRNAs move relative to the large subunit, from the classical A/A and P/P binding states into hybrid A/P and P/E states (Fig. 1b). In the second step, the mRNA and the tRNA anticodon stem-loops (ASLs) move relative to the small subunit, which allows the tRNAs to adopt classical P/P and E/E conformations (Fig. 1c). While hybrid-state formation occurs spontaneously^9^, ASL movement is significantly influenced by the presence of elongation factor G (EF-G)^10^. Furthermore, tRNA movement between the binding sites is associated with internal motions of the ribosome, including rotations of the 30S body and head domains (Fig. 1d). Specifically, the 30S body rotates relative to the 50S subunit (intersubunit rotation) during hybrid-state formation^7^,^11^, whereas rotation of the 30S head (also known as ‘head swivel') is associated with the movement of mRNA and tRNA on the small subunit^12^,^13^,^14^.

It has been accepted that Brownian noise is a major contributor to conformational changes in the ribosome during elongation^15^. As a result, the study of the ribosome is now expanding to questions about the ribosome's energy landscape. The energy landscape perspective has the broad utility of allowing one to quantitatively explore the structural and energetic factors that govern biomolecular dynamics. While these tools are well established for describing biomolecular folding and function^16^,^17^,^18^,^19^,^20^,^21^, the development of methods to quantitatively analyse the ribosome's landscape^22^,^23^,^24^,^25^,^26^ is still in its infancy. One avenue for exploring the landscape is through molecular dynamics simulations. At the highest level of detail, quantum mechanical models can provide insights into catalytic steps^27^,^28^,^29^. Similarly, classical mechanical models with explicit-solvent representations are often used to predict the enthalpic contributions of intermolecular interactions^26^,^30^,^31^. While free energies for the full ribosome are currently not accessible with explicit-solvent models, the free energy can be calculated for smaller RNAs^32^, or for isolated regions of the ribosome, such as the decoding centre^33^,^34^. It is important to note that, while these explicit-solvent models are continuously being refined for RNA^35^, it is not yet clear which model provides the most accurate description of RNA dynamics, in general^36^. To complement the detailed pictures obtained from explicit-solvent calculations, coarse-grained elastic network models can describe collective, correlated motions of the ribosome. A key finding from coarse-grained models has been that the architecture of the ribosome intrinsically favours global rearrangements along specific directions^37^,^38^,^39^. Since such a vast range of dynamical processes are associated with the ribosome, it is apparent that an equally wide range of models will be needed to fully characterize the numerous contributors to function.

Here we use a model that employs an intermediate level of resolution, in order to probe the physical relationship between mRNA–tRNA movement and 30S head motions during the second step of translocation (30S translocation). Specifically, we apply molecular dynamics simulations with an all-atom structure-based model^40^, and identify the roles of tRNA/ribosome sterics and flexibility during this large-scale rearrangement. In this model, the unrotated conformation of the ribosome corresponds to the potential energy minimum. In addition, each tRNA molecule has affinity for two adjacent binding sites on the ribosome, where classical tRNA binding conformations are potential energy minima. With this model, we simulated 250 translocation events, which provide a description of sterically accessible pathways and intermediate ensembles between the A/P–P/E and P/P–E/E ensembles. While the potential energy minima are defined entirely by the unrotated, classical conformations of the ribosome and tRNA, these simulations capture the known chimeric ap/P–pe/E intermediate, where the 30S head is highly rotated. This head-rotated intermediate is in excellent agreement with numerous structures of the ribosome trapped in mid-translocational conformations^12^,^13^,^14^,^41^. Our simulations also predict the presence of a previously unreported intermediate ensemble that is characterized by a distinct tilt of the 30S head. To identify the structural features that contribute to the head-tilted ensemble, we perform hundreds of additional simulations in which specific residues in the 30S subunit are perturbed. These perturbations may be considered a computational analogue of experimental mutational analysis. Using these perturbed models, we show that the tilt motion of the head is a direct consequence of steric interactions between the ribosome and tRNA. Together, our results demonstrate how a simple energetic model can be used to partition the contributors to dynamics, thereby revealing how molecular structure gives rise to biological function.

## Results

To elucidate the interplay between 30S head dynamics and tRNA displacement on the small subunit, we performed molecular dynamics simulations of the full 70S ribosome in complex with two tRNA^Phe^ molecules, mRNA and EF-G. For these simulations, we used a multi-basin all-atom structure-based model^40^. In these models, potential energy minima are defined based on (usually 2–3) experimentally derived structures. Here we used crystallographic structures of unrotated ribosomes to construct a potential energy landscape for translocation (see Supplementary Methods for details), where each tRNA molecule can form short-range (∼10 Å) stabilizing interactions with two adjacent binding sites on the ribosome (A and P sites, or P and E sites). By construction, the classical tRNA binding conformations and the unrotated ribosome conformation correspond to potential energy minima. In addition, the intramolecular interactions in EF-G were defined such that the ribosome-bound post-translocational (POST) conformation^42^ corresponds to the global potential energy minimum. This overall energetic representation is consistent with X-ray crystallography and cryo-electron microscopy (cryo-EM) results. That is, the unrotated and classical conformations are minima in the modelled energy landscape, which is necessarily the case in solution since these conformations may be structurally resolved.

Using our structure-based model, we first simulated 250 independent, unrestrained (unguided) translocation events. While our focus is on the dynamics that occur after the tRNAs adopt the A/P and P/E binding conformations, we initiated simulations from a model representing an earlier point in the translocation process. Specifically, each simulation was initiated with the A- and P-site tRNAs in A/A and P/E binding positions (Fig. 2a,b; models described in ref. 43). By starting the simulations from this conformation, we considered the initial A/A–P/E to A/P–P/E transition as an equilibration period. This equilibration step was provided, in order to reduce the influence of initial conditions on the dynamics that follow the adoption of the A/P–P/E state. Each simulation was terminated once the ribosome reached the POST state, where the tRNAs adopt the classical P/P and E/E binding conformations. The structural characteristics of all simulated events are qualitatively similar, where the A site tRNA first moves relative to the large subunit, resulting in the formation of the A/P–P/E conformation (Fig. 2c,d and Supplementary Fig. 1). Then the ASLs of the tRNAs move relative to the small subunit, which leads to the formation of the P/P–E/E conformation. As noted above, rearrangements of the A-site tRNA that occur before reaching the hybrid A/P–P/E conformation were not analysed. Throughout the A/P–P/E to POST transition, EF-G maintains a post-translocation-like conformation (Fig. 2e and Supplementary Fig. 2). Despite the lack of a conformational rearrangement in EF-G after the A/P–P/E stage (Fig. 2f–h), EF-G may still facilitate subsequent translocation substeps. That is, since domain IV of EF-G is in close proximity of the 30S A site, it may serve as a steric ‘doorstop' that prevents reverse tRNA motion, which would favour forward translocation.

In contrast to previous simulations of tRNA hybrid-state formation^44^, targeting protocols were not used to guide the dynamics in the current study. Rather, in the unrestrained simulations here, each transition occurred spontaneously (that is, stochastically). To illustrate the differences between unrestrained and targeted dynamics, we also performed 100 targeted molecular dynamics (TMD) simulations of translocation (Supplementary Fig. 3 and Methods). When targeted molecular dynamics was employed, the dynamics deviate significantly from that observed in the unrestrained simulations. As discussed below, the stochastic events from unguided simulations reveal how steric interactions between the ribosome and tRNA can lead to rotational and tilt movements of the 30S head during the second step of translocation.

### Sterics and flexibility give rise to a known intermediate

The dynamics of biomolecular function are governed by the free-energy landscape, which is a result of stabilizing interactions, entropy and steric repulsion. While in a structure-based model the dominant potential energy minima are defined *a priori*, a more complex free-energy landscape can arise from entropic contributions and steric restrictions on tRNA/ribosome movement. As an example, simulations of aa-tRNA accommodation have shown that structural effects lead to pronounced free-energy barriers, even when the potential energy surface is smooth^25^. Since the current simulations were terminated once the POST state was reached, the free energy is not directly accessible here. However, one may infer the structural characteristics of likely intermediates and transition states from these simulations. Specifically, when the ribosome encounters a free-energy barrier, molecular movements are transiently impeded, which leads to increased sampling of the local configuration space. Hence, a poorly populated region between two highly sampled regions is the signature of a free-energy barrier (a transition state) that separates two minima (intermediates or endpoint ensembles).

To identify intermediates associated with translocation, we calculated probability distributions as functions of structural coordinates. These distributions represent the statistical properties of the dynamics, obtained from hundreds of independent simulated events. We characterize the movement of the 30S head during mRNA–tRNA translocation, by calculating the probability as a function of the ASL position of the P-site tRNA (relative to the 30S body) and the rotation of the 30S head: *P*(*R*_P−ASL_, *φ*_head_) (Fig. 3a). See Supplementary Methods for detailed descriptions of tRNA and ribosome coordinates. To provide a complementary description, we also calculated the probability as a function of the ASL position of the P-site tRNA and the magnitude of head tilt: *P*(*R*_P−ASL_, *θ*_head_) (Fig. 3b). In these distributions, there are two visibly distinct populations separating the A/P–P/E and P/P–E/E ensembles, which are labelled ap/P–pe/E and HT (for head-tilted). Populating the ap/P–pe/E ensemble is associated with movement of the tRNA from the P site towards the E site of the 30S body (*R*_P−ASL_≈4–6 Å) and a large rotation of the 30S head (*φ*_head_=16.5±1.5°;mean±s.d.). For reference, *R*_P−ASL_=0 when the tRNA is in the 30S E site. After sampling the ap/P–pe/E enemble, the system transitions to the head-tilted ensemble, which involves nearly complete back rotation of the head (Δ*φ*_head_≈−13°). Interestingly, this reverse head rotation is accompanied by a previously unreported tilt of the 30S head (*θ*_head_=9.5±2.2°) (Fig. 3b), which results in displacement of the head away from the 30S–50S intersubunit interface. The translocation process is completed when the tRNA molecules reach the POST state, where the head rotation and tilt angles (*φ*_head_ and *θ*_head_) return to near-zero values.

The ap/P–pe/E ensemble observed in our simulations is in excellent agreement with models derived from cryo-EM and X-ray crystallography^12^,^13^,^14^,^41^ (Supplementary Table 1). In particular, the simulated ap/P–pe/E ensemble is most similar to the cryo-EM reconstruction reported in ref. 12, which describes the ribosome with EF-G bound and tRNAs in ap/P and pe/E binding positions. Before comparing this model with our intermediate ensemble, it is instructive to discuss the overall characteristics of the cryo-EM model. In the ap/P–pe/E ribosome, the tRNA molecules are partially displaced relative to the 30S body, and the 30S head is highly rotated. This configuration allows the tRNAs to contact the A and P sites of the head, while simultaneously contacting the P and E sites of the body. This chimeric form of intrasubunit binding is reflected in the naming convention, as introduced previously^14^: pe/E indicates that the P-site tRNA contacts the P site of the 30S head (p), the E site of the 30S body (e), and the E site of the 50S subunit (E).

Since a given experimental structure represents the average configuration within a sample, we calculated the average structure of all simulated snapshots that were in the ap/P–pe/E ensemble. The root-mean-square deviation (RMSD) between the average ap/P–pe/E configuration and the cryo-EM model^12^ is 1.93 Å (calculated for all core atoms, see Supplementary Methods). While this average structure provides an overall description of the ensemble, the averaging process yields a simplified perspective that suppresses information about fluctuations. Accordingly, subsequent comparisons of structural metrics include the mean and s.d. of each coordinate, calculated for a specified ensemble. In terms of subunit rotations, our ap/P–pe/E ensemble is characterized by a large rotation of the head (*φ*_head_=16.5±1.5°; Fig. 3a) and a modest degree of body rotation (*φ*_body_=1.9±1.2°; Supplementary Fig. 4). These values agree well with those obtained from cryo-EM and X-ray models^12^,^13^,^14^,^41^, particularly with those of ref. 41, for which *φ*_head_=16.6° and *φ*_body_=1.6° (see Supplementary Table 1 for rotation angles calculated for other experimental structures). Consistent with the experimental structures mentioned above, we also find that EF-G adopts a post-translocation-like conformation, where its domain IV extends towards the A site of the 30S subunit. Specifically, the average RMSD of EF-G in the ap/P–pe/E ensemble from its POST conformation is 2.2±0.3 Å (Supplementary Fig. 2). Furthermore, in the simulated ap/P–pe/E ensemble, there is a compaction of the ASLs of the tRNAs. To describe this compaction, we calculated the distance between the P atoms of the A31 residues in the A- and P-site tRNAs: *R*_A31_ (Supplementary Fig. 5a). When the tRNA molecules are in classical binding positions, *R*_A31_ is 30.0 Å (A/A–P/P) and 25.2 Å (P/P–E/E)^45^. In contrast, in the ap/P–pe/E intermediate from cryo-EM^12^, *R*_A31_ adopts a smaller value of 21.3 Å. This experimentally observed compaction of the ASLs is very similar to that found in our simulated ap/P–pe/E ensemble, where *R*_A31_=19.1±1.6 Å (Supplementary Fig. 5b). As a final point of comparison, the ASL position of the P-site tRNA relative to the 30S head is also consistent with the experimentally obtained structures of this intermediate^12^,^13^,^14^,^41^. Specifically, the distance between the P atoms of A31 in the P-site tRNA and A1229 in the 16S rRNA (*R*_pe_) is 12.2 Å in the structural model^12^, and *R*_pe_=11.5±1.3 Å in the simulated ap/P–pe/E ensemble (Supplementary Table 1). Overall, the high level of agreement between our simulated ap/P–pe/E ensemble and the models derived experimentally demonstrates that the simplified energetic model is sufficient to capture this en-route translocation intermediate.

After the ap/P–pe/E ensemble is reached, the simulated system populates another intermediate ensemble, the head-tilted ensemble (labeled HT in Fig. 3a,b). The most striking structural feature of this ensemble is that the 30S head is highly tilted (*θ*_head_=9.5±2.2°, Fig. 3b), which has not been reported in the context of translocation. However, a similar degree of head tilting has been observed in the context of transfer-messenger RNA rescue^46^ (*θ*_head_=11.5°). Below, we discuss the structural and energetic features that give rise to this large-scale collective tilt motion, as well as the degree to which tilting is correlated with head rotation and tRNA displacement.

### mRNA–tRNA translocation involves 30S head rotation and tilt

**Figure 1:**
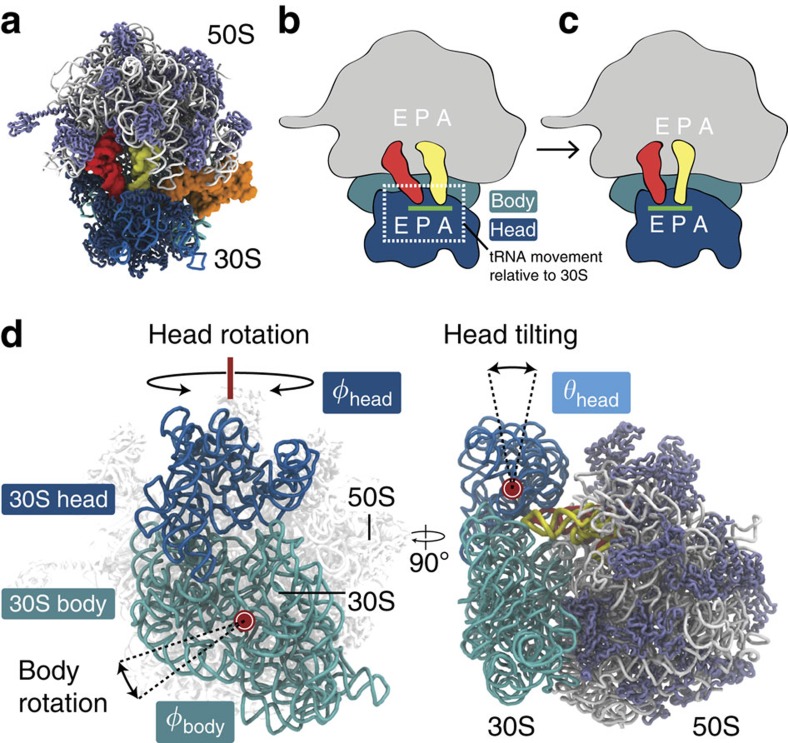
Structural description of translocation on the 30S subunit. (**a**) Structure of the 70S ribosome with two tRNAs (red and yellow) and EF-G (orange). The 23S mRNA and 50S proteins are shown in white and ice blue. The 16S mRNA and 30S proteins are in blue and dark blue. (**b**) Schematic of the ribosome with mRNA (green) bound on the 30S subunit, and the P- and A-site tRNAs in hybrid P/E (red) and A/P (yellow) conformations. The 50S subunit is depicted in grey. The head and body domains of the 30S subunit are shown in blue and cyan. The region in which mRNA–tRNA movement occurs on the 30S is demarcated by a dashed box. (**c**) Schematic of tRNAs in classical E/E and P/P conformations, after translocation on the 30S subunit. (**d**) Subunit rotations are described by: *φ*_body_ (body rotation), *φ*_head_ (head rotation) and *θ*_head_ (magnitude of head tilting). Counterclockwise rotation of the body (from the perspective shown) and counterclockwise rotation of the head (viewed from above) are defined as positive. All structural representations were prepared using visual molecular dynamics (VMD)^65^.

During the transition from the ap/P–pe/E to the head-tilted ensemble, there is an apparent correlation between back rotation and tilting of the 30S head (Δ*φ*_head_≈−13° in Fig. 3a, and Δ*θ*_head_≈6° in Fig. 3b). To quantify the magnitude and direction of head tilting, we used the angles *θ*_head_ and *χ*_head_ (shown schematically in Figs 1d and 3c; for further details see Supplementary Figs 6 and 7, and Supplementary Methods). The tilt direction (*χ*_head_) is the orientation of the axis about which tilting occurs. We defined *χ*_head_=0 such that it corresponds to tilting that is approximately about the axis of the mRNA backbone, where the 30S head is displaced away from the 30S–50S interface (Fig. 3c, and Supplementary Figs 6 and 7). In addition to the direction of tilting, the magnitude of head tilting is described by *θ*_head_, as noted above. To characterize the direction of head tilting in the head-tilted ensemble (for example, parallel or perpendicular to the mRNA), we calculated the probability *P*(*χ*_head_) for head-tilted conformations (Fig. 3d). Since there were no explicit restrictions on the direction of tilting, it would be conceivable that tilting could occur in directions that are parallel or perpendicular to the axis of the mRNA. However, we find that *P*(*χ*_head_) peakes at *χ*_head_=0, where the full-width at half-maximum is 24°. This narrow distribution of tilt-axis direction demonstrates that tilting in the head-tilted ensemble occurs predominantly about the mRNA axis.

The apparent balance between rotation and tilt in the head-tilted ensemble can be interpreted as enabling ‘opening' of the mRNA binding track on the 30S subunit. This is consistent with previous structural studies that have found substantial changes in the steric features of the mRNA binding track upon head rotation^47^. As discussed in ref. 47, residues G1338 to U1341 in the head (that is, the ‘PE loop') sterically separates the P and E sites of the 30S subunit. This steric obstacle has been described as a gate that opens when the PE loop (in the head) is displaced relative to residue A790 (in the body)^47^. Here to measure gate opening, we used the distance between the P atoms of residues U1340 and A790: *R*_gate_ (Fig. 4a). In a crystallographic model of the classical conformation, where the 30S head is unrotated and untilted, *R*_gate_=16.7 Å (ref. 45). In contrast, in the ap/P–pe/E structural model^12^, where the head is highly rotated, *R*_gate_ has a significantly larger value of 25.1 Å. This substantial change in the gate region has led to the argument that opening may allow tRNA molecules to transition to the POST conformation^4^,^6^. While gate dynamics likely contributes to translocation, the fact that the head-rotated ap/P–pe/E conformation is stable when the gate is open^12^,^13^ suggests that head rotation alone is not sufficient to confer tRNA passage.

To describe the gate dynamics in the simulations, we evaluated the probability as a function of the ASL position of the P-site tRNA and the opening of the gate: *P*(*R*_P−ASL_, *R*_gate_) (Fig. 4b). Consistent with the notion that gate-opening facilitates translocation, we observe a large degree of gate opening (*R*_gate_=27.7±1.1 Å) in the ap/P–pe/E ensemble. Similarly, we find that gate separation remains large in the head-tilted ensemble (*R*_gate_=24.5±1.5 Å). This is particularly interesting because, if the head were unable to tilt, one would expect an approximately linear relationship between head rotation (*φ*_head_) and gate opening (*R*_gate_). However, during the transition from the ap/P–pe/E to the head-tilted ensemble, we find a marginal change in gate separation (Δ*R*_gate_≈−3 Å) over a large range of head rotation angles (Δ*φ*_head_≈−13°). In contrast, during the subsequent transition from the head-tilted to the P/P–E/E ensemble, there is a significant reduction in gate separation (Δ*R*_gate_≈−7 Å) over a smaller range of rotation values (Δ*φ*_head_≈−5°). While initial opening of the gate is associated with head rotation, as implicated by structural data^12^,^13^,^14^,^41^, our simulations predict that gate closure only occurs when the head relaxes from a tilted orientation. This suggests an extended description of gate opening, where there are compensatory rotation and tilting fluctuations that together provide a sufficiently wide corridor for tRNA to translocate.

### Isolating the origins of head tilting

From visual inspection of the simulated trajectories, it is apparent that the PE loop and protein S13 transiently interact with the tRNA molecules during translocation (Fig. 5a–c and Supplementary Movie 1). Consistent with this observation, we verified that in all simulations the tRNAs transiently contact (are within 3 Å of) the C-terminal tail of S13 and the PE loop. To evaluate the extent to which these tRNA–ribosome interactions contribute to head tilting, we performed additional simulations in which specific steric interactions were removed. Specifically, we tested the relative influence of protein S13 and the PE loop by excluding each of their steric contributions in separate sets of simulations. For these tests, we used the following variations of our multi-basin structure-based model: Model (1) Same model as described above; Model (2) Identical to Model 1, except steric interactions between S13 and tRNA were not included; Model (3) Identical to Model 1, except steric interactions between the PE loop and tRNA were not included; Model (4) Identical to the above models, except both S13 and PE loop sterics were not included. See Supplementary Methods for technical descriptions of the models. These simulations mimic an ideal mutation experiment. That is, mutational studies aim to modulate a single variable at a time, in order to elucidate the relative contributions of specific interactions. Here we removed specific steric interactions between residues without introducing indirect perturbations. Thus, changes in the dynamics can be directly attributed to the steric interactions that are varied.

For each of the four models, we simulated ∼200 independent, unrestrained translocation events. To compare the degree of head titling in each model, we calculated the probability as a function of head rotation and head tilting: *P*(*φ*_head_, *θ*_head_) (Fig. 5d–g). Since the focus of this exercise was to explore the role of specific interactions during formation of the head-tilted ensemble, these additional simulations were initiated from an ap/P–pe/E conformation and terminated when the system adopted the P/P–E/E conformation (*φ*_head_≈0° and *θ*_head_≈0°). We find that steric interactions have significant effects on the degree of head tilting, as highlighted by the average tilt angle as a function of head rotation: 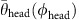 (dashed lines in Fig. 5d–g). When all steric interactions are included (Model 1), 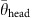 reaches a maximum value of ∼10°. When the steric interactions between protein S13 and tRNA are excluded (Model 2), the maximum value of 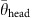 is reduced to ∼8°. In contrast, there is a more significant reduction in head tilting when the sterics of the PE loop are not included (Model 3). Under those conditions, 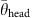 only reaches a value of ∼6°, corresponding to a 40% reduction in the scale of head tilting. Finally, when the sterics of both S13 and the PE loop are not included (Model 4), head tilting is further attenuated with a maximum 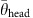 value of only 3–4°. This residual head tilting indicates that, while the dominant contributions stem from PE loop and S13 interactions, additional structural factors also influence tilting. Together, comparison of the models demonstrates that both the PE loop and protein S13 contribute to head tilting during back rotation of the head, though the relative contribution of the PE loop is larger.

Previous biochemical and genetic assays have shown that protein S13 is involved in stabilizing the pre-translocational state^48^, and that ribosomes lacking S13 exhibit increased rates of factor-free translation^49^. Consistent with these observations, our results suggest that protein S13 (in addition to the PE loop) poses a steric barrier that impedes the movement of the mRNA–tRNA complex towards the POST state. That is, before the reverse rotation of the 30S head (before, and including the ap/P–pe/E state), the C terminus of S13 is positioned between the A- and P-site tRNAs (Fig. 5a). As discussed above, this positioning of S13 then leads to head tilting during reverse rotation. It is conceivable that the steric barrier introduced by S13 may also impede forward movement of the A-site tRNA relative to the 30S head. This suggests that, in addition to potentially introducing stabilizing interactions between S13 and tRNA, the steric influence of S13 may increase the observed dwell time of the pre-translocational state.

## Discussion

As our physical–chemical understanding of the ribosome continues to be developed, an emerging theme is that elongation dynamics result from an interplay between energy accumulation and release in the ribosome and tRNA. In the words of Frank *et al.*^50^, a tRNA molecule may be regarded as a (non-linear) ‘molecular spring', where the classical tRNA conformations correspond to low-energy states. During elongation, tRNA molecules then interconvert between nonclassical states, which is facilitated by tRNA–ribosome interactions and factor binding. Consistent with this perspective, our model describes the classical conformations of tRNA as energetic minima. That is, when a tRNA adopts hybrid conformations, the internal energy of the molecule is higher (∼5 reduced units, Supplementary Methods), though these states are also stabilized by ribosome–tRNA interactions. In terms of concepts developed to describe protein function^51^, nonclassical binding states are associated with an accumulation of ‘strain energy', which is released when the system reaches the low-energy P/P–E/E conformation.

The strain energy framework may also be applied to the ribosome. Specifically, when the tRNA–ribosome assembly reaches the chimeric ap/P–pe/E ensemble, the 30S head becomes highly rotated, implying that the small subunit is in a strained state. In contrast, in the ap/P–pe/E ensemble, the tRNAs adopt conformations that are near their classical P/P and E/E conformations. That is, the RMSD values of the average simulated ap/P and pe/E tRNA configurations, relative to the P/P and E/E conformations, are only 0.42 and 0.96 Å. Experiments have shown that thermal energy enables tRNA to transiently sample energetically strained hybrid conformations^9^,^11^,^52^. Our analysis extends this description, and suggests that strain energy accumulated in the tRNAs may be subsequently transferred to the ribosome as the ap/P–pe/E ensemble is reached. This strain in the ribosome is then released as the system relaxes to the unrotated POST state.

With regards to strain in EF-G, structural studies have implicated a range of different conformations of the factor bound to ribosomes in numerous states, including the A/A–P/P^53^, A/P*–P/E^54^, ap/ap–pe/E^13^ and ap/P–pe/E^12^ states. These structural considerations suggest that strain accumulation and release in EF-G may likely occur during earlier stages of translocation, particularly before the system reaches the head-tilted ensemble. In the preceding ap/P–pe/E ensemble, EF-G adopts a post-translocation-like conformation, where its domain IV is adjacent to the ap/P–tRNA (Supplementary Fig. 2), implying that EF-G is energetically relaxed. Therefore, as the ribosome transitions from the ap/P–pe/E via the head-tilted to the P/P–E/E ensemble, strain energy is not expected to be significant. Rather, during these rearrangements, the presence of close interactions between domain IV and tRNA suggests that EF-G may act in a more passive capacity. That is, it appears to simply serve as a steric doorstop that prevents reverse movement of the tRNAs. By preventing reverse motion, EF-G effectively stabilizes head-rotated conformations, relative to the pre-translocational state, as suggested by ref. 13. In addition, compaction of the tRNA ASLs and domain IV may position the tRNA molecules such that the free-energy barrier for forward translocation is reduced. This raises the possibility that EF-G may accelerate translocation kinetics, even in the absence of direct energy transfer between GTP hydrolysis and the ribosome.

The picture of strain accumulation and release provides a framework for exploring the balance between molecular flexibility and sterics, which may be extended to account for more detailed energetic contributions. While our models elucidate steric effects and implicate correlated fluctuations, the complete energy landscape of the ribosome is complex, and it results from many factors, including charge–charge interactions and solvation. In the current model, the stabilizing contribution of these factors are implicitly described. However, when the system is far from the endpoints (for example, in transition states), many transient interactions are formed. Here we have shown that steric interactions frequently occur during translocation, primarily in the form of RNA–RNA backbone interactions. Since the RNA backbone is negatively charged, one could expect that the observed steric effects will be amplified by electrostatic effects. For example, the steric repulsion between the P-site tRNA and the PE loop will likely become stronger when explicit electrostatics are included, though counterion effects may partially mitigate this amplification. Similarly, the degree of tRNA compaction in the ap/P–pe/E ensemble may also be modulated by electrostatic interactions. While the current model predicts a compaction of the tRNA ASLs that is similar to cryo-EM observations, the tRNA molecules are roughly 1 Å closer to each other in the simulations. Repulsive charge–charge interactions between the tRNAs may decrease the degree of compaction, which would further improve the level of agreement between cryo-EM and the simulations. In addition to electrostatics, the models may be extended to explore the role of desolvation. In the context of protein folding, many theoretical and experimental findings have implicated an essential role of desolvation^20^,^55^,^56^,^57^, where models that include desolvation effects predict more cooperative protein folding dynamics. In terms of RNA base stacking, desolvation effects have been extensively quantified computationally^58^. Since the conformational rearrangements probed in the current study do not involve breaking or formation of base–stacking interactions, we do not expect the current results to be sensitive to desolvation effects. However, for other steps of elongation, such as displacement of the tRNA CCA end inside the peptidyl transferase centre, the effect of desolvation may be pronounced.

In summary, this work demonstrates how simple energetic considerations can elucidate the relationship between molecular structure and functional dynamics in the ribosome. Our findings provide a structural and energetic framework for describing and interpreting the complex dynamics of mRNA–tRNA translocation. With this framework, we may now explore the physical relationship between the many molecular components of the ribosome, which can help guide the design of more precise experimental measurements. Through the continued integration of theoretical and experimental insights, it will be possible to establish a cohesive physical–chemical description that bridges detailed biochemical interactions and large-scale dynamics.

## Methods

### The forcefield

To simulate translocation, we used a multi-basin all-atom structure-based model. In the simplest form, a single-basin structure-based model defines an experimentally derived structure as the global potential energy minimum^40^,^59^. To construct a potential energy function for translocation, we first generated a structure-based model for the P/P–E/E configuration. In addition, contacts between the ribosome and mRNA–tRNA, which are found in the A/A–P/P configuration were included as stabilizing interactions. The energetic weights of the contacts between the ribosome and mRNA–tRNA were rescaled by a factor of 0.3, to account for the transient nature of mRNA–tRNA binding. That is, intrasubunit interactions maintain the long-timescale structural integrity of the ribosome, whereas mRNA–tRNA associates and dissociates from the ribosome on much shorter timescales. Interface contacts between the 30S and 50S subunits, as well as between the 30S head and 30S body, were also weakened (see Supplementary Methods for details). These contacts were reduced in strength to mimic the observation that subunit rotations spontaneously occur^11^ on timescales that are far shorter than the lifetime of a fully formed ribosome. For complete details, see Supplementary Information.

### Simulation details

**Figure 2:**
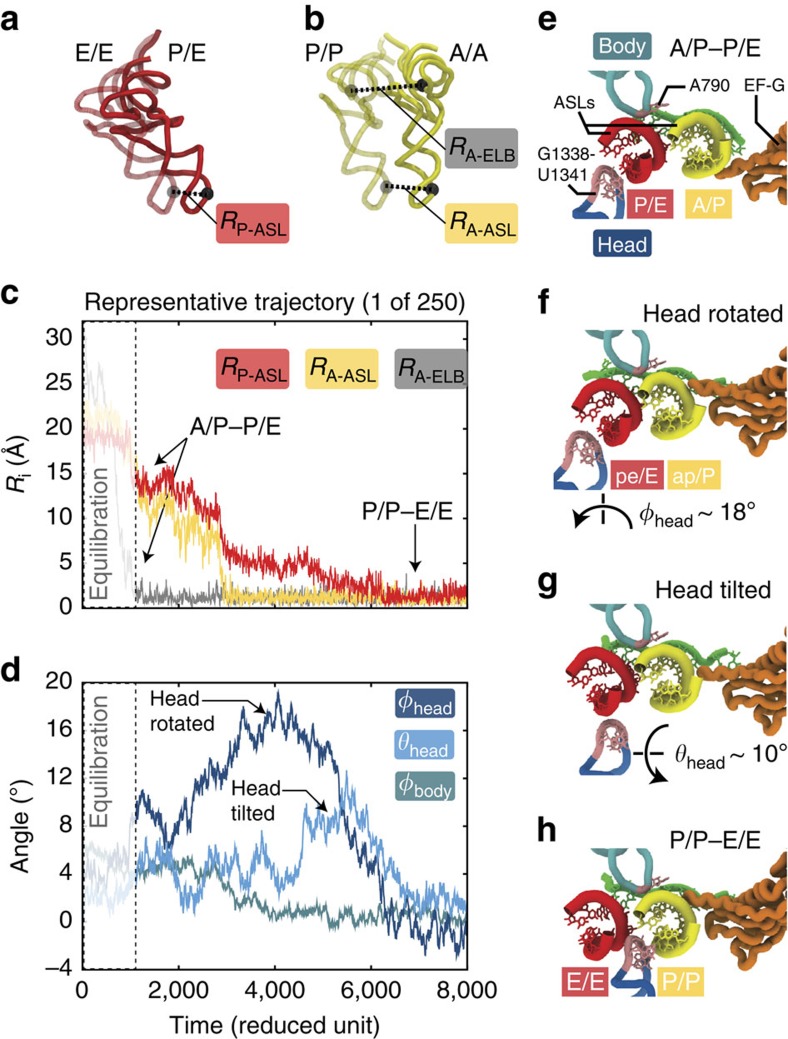
Complex 30S translocation dynamics emerge from a simple energetic model. (**a**,**b**) Multiple coordinates are used to describe tRNA movement. *R*_P−ASL_ and *R*_A−ASL_ are distances of the anticodon stem-loops (ASLs) of the P and A site tRNAs (red and yellow) to their E/E and P/P positions. These ASL distances were calculated after 30S body alignment of the simulated trajectory to the POST conformation (see Supplementary Information for details). *R*_A−ELB_ is the distance of the A-tRNA elbow to its location when in the P/P conformation. Elbow distance is determined after 23S alignment of the trajectory to the POST conformation. In **a** and **b**, the P/E and A/A tRNA conformations (initial configuration in the simulation) are shown as opaque. The endpoint E/E and P/P conformations are in ghost representation. (**c**,**d**) Representative time traces for a single simulated event (1 of 250) shows large-scale movements of the tRNAs (20–30 Å, **c**) and transient subunit rotations in the ribosome (**d**). In all 250 simulations, formation of the A/P–P/E conformation, where *R*_A−ELB_ (grey line) decreases from ∼30 to ∼0 Å, precedes displacement of the ASLs (red and yellow lines). In **c** and **d**, the dashed white-faded boxes outline the initial equilibration period, where the A-site tRNA elbow relaxes to the 50S P site and adopts an A/P conformation. (**e**–**h**) Structural snapshots of the mRNA binding track (from the perspective of the 50S subunit) during translocation illustrate the tRNA ASL positions, relative to the 30S body and head. During the initial equilibration period, the system adopts an A/P–P/E conformation (**e**). During translocation, the 30S head rotates (**f**), which is followed by back rotation and tilting of the head (**g**). Translocation is completed when the classical P/P–E/E conformation (**h**) is reached.

**Figure 3:**
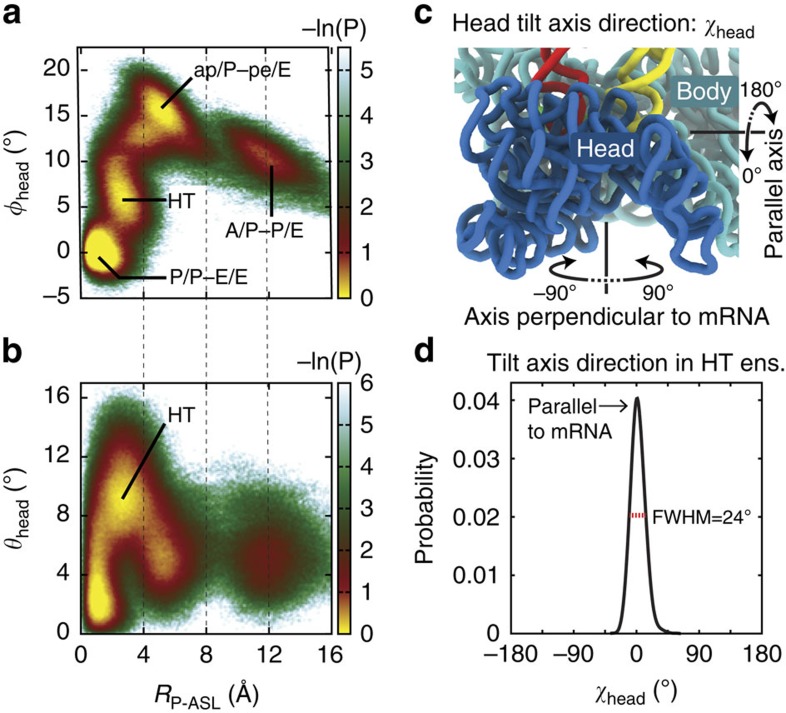
A/P–P/E to P/P–E/E transition involves large-scale rotation and tilting of the 30S head. Probability distributions calculated from 250 unrestrained (unguided) simulations highlight the relationship between tRNA and 30S head motions. (**a**) For the transition from the A/P–P/E to the P/P–E/E ensemble, the probability *P*(*R*_P−ASL_,*φ*_head_) indicates the presence of two intermediate ensembles: a chimeric ap/P–pe/E ensemble and a head-tilted (HT) ensemble. The ap/P–pe/E ensemble includes highly rotated head configurations (*φ*_head_≈14–20°). Adopting the HT ensemble is associated with nearly complete back rotation of the head (Δ*φ*_head_ ≈−13°). (**b**) *P*(*R*_P−ASL_,*θ*_head_) shows that there is a large degree of head tilting (*θ*_head_≈10°) in the HT ensemble. (**c**) Representative directions (parallel or perpendicular to the mRNA) of the tilt axis of the 30S head are shown. The direction of the tilt axis is measured by *χ*_head_ (see Supplementary Information for details). By construction, *χ*_head_=0° corresponds to tilting about an axis that is parallel to the mRNA, where the head is displaced away from the 30S–50S interface. Similarly, *χ*_head_=180° corresponds to tilting towards the 30S–50S interface. In contrast, *χ*_head_=±90° corresponds to tilting that is perpendicular to the mRNA. (**d**) *P*(*χ*_head_) shows that head tilting in the HT ensemble occurs predominantly along the *χ*_head_=0° direction (that is, about the mRNA axis), with a full-width at half-maximum (FWHM; red bar) of 24°. As a technical note, simulations were initiated from an A/A–P/E conformation. Initial relaxation into the A/P–P/E ensemble was allowed before data was included for analysis. Accordingly, probability distributions were calculated for *R*_A−ELB_<4 Å.

**Figure 4:**
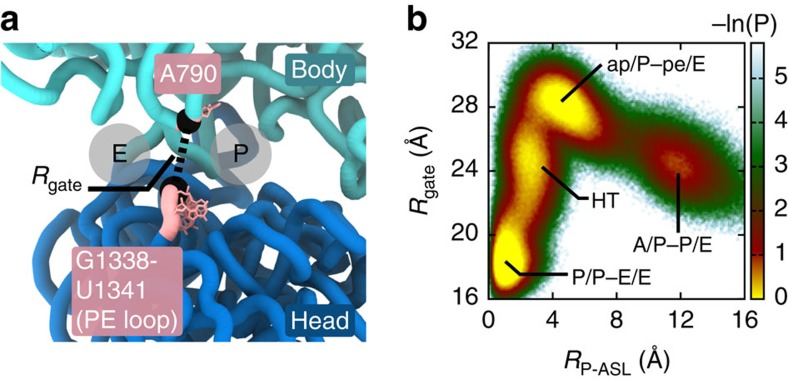
Changes in steric features of the mRNA binding track are associated with tRNA movement. (**a**) The PE loop (residues G1338-U1341) in the head and residue A790 in the body (pink) form a steric gate separating the E and P sites. *R*_gate_ is the distance between the P atoms of U1340 and A790 (black beads), and is used to probe opening and closing of the gate. (**b**) The probability distribution *P*(*R*_P−ASL_,*R*_gate_) implicates a large degree of gate opening (*R*_gate_=26–30 Å) in the ap/P–pe/E ensemble, which is associated with head rotation (*φ*_head_≈18°, cf. Fig. 3a,b). In the subsequently populated HT ensemble, gate opening is only slightly decreased. While there is a reduced degree of head rotation in the HT ensemble, large gate opening (*R*_gate_≈23–26 Å) is maintained by a compensatory tilt of the head (*θ*_head_≈10°, cf. Fig. 3a,b).

**Figure 5:**
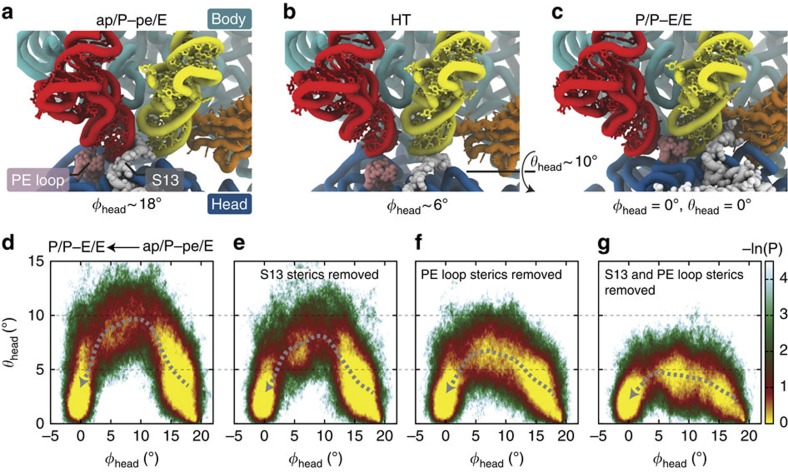
Head tilting results from tRNA interactions with the PE loop and protein S13. Representative structural snapshots of (**a**) ap/P–pe/E, (**b**) HT and (**c**) P/P–E/E configurations. The PE loop (pink) and protein S13 (grey) are in close proximity to the P- and A-site tRNAs (red and yellow) during translocation. To elucidate the extent to which the PE loop and protein S13 contribute to tilting, perturbations were introduced to the model. (**d**–**g**) Probability distributions *P*(*φ*_head_, *θ*_head_) illustrate the relative influence of steric composition on tilting. When all sterics are included (**d**), 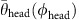 (the average tilt as a function of rotation; dashed grey line) reaches a maximum value of ∼10°. When steric interactions between protein S13 and tRNA are not included (**e**), the maximum value of 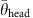 is reduced to ∼8°. When the sterics of S13 are present, while steric interactions between the PE loop and tRNA are not included (**f**), the maximum tilt is reduced to ∼6°. When both S13 and PE loop sterics are not included (**g**), head tilting is most strongly attenuated (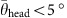).

Simulations were performed with the Gromacs (v4.6.1) software package^60^,^61^ using forcefield files generated by the SMOG-model web server (smog-server.org)^62^. Reduced units were used for all calculations. Each simulation was performed for a minimum of 4 × 10^6^ time steps of size 0.002, and was extended until the P/P–E/E configuration was adopted. Employing a timescale correction factor reported by Kouza *et al.*^63^, the effective timescale of each simulation may be roughly approximated as 10–100 μs. For a detailed discussion on timescale estimates in all-atom structure-based models, see ref. 25 and the Supplementary Material of ref. 64. Langevin Dynamics protocols were used to ensure a constant temperature of 0.5 (reduced units). As discussed elsewhere^64^, the scale of structural fluctuations at this temperature is consistent with that obtained using an explicit-solvent model at 300 K, and with values estimated from crystallographic B-factors. 250 simulations were initially performed for the full mRNA–tRNA translocation process (results presented in Figs 2, 3, 4). Around 200 additional simulations were performed for each of the four modified forcefields, where the sterics of the PE loop and/or protein S13 were excluded (results presented in Fig. 5). In total, 1,031 translocation events were simulated without the use of targeting or steering protocols.

## Additional information

**How to cite this article**: Nguyen, K. and Whitford P. C. steric interactions lead to collective tilting motion in the ribosome during mRNA–tRNA translocation. *Nat. Commun.* 7:10586 doi: 10.1038/ncomms10586 (2016).

## Supplementary Material

Supplementary Information Supplementary Figures 1-8, Supplementary Table 1, Supplementary Methods and Supplementary References

Supplementary Movie 1 Representative trajectory of mRNA-tRNA translocation highlighting 30S head tilting during back rotation.
