# Supplementary material for: Steric interactions lead to collective tilting motion in the ribosome during mRNA–tRNA translocation
Source: Nat Commun. 2016 Feb 3;7:10586. doi: 10.1038/ncomms10586 (PMC4742886; doi:10.1038/ncomms10586)
Supplement: Supplementary Information — Supplementary Figures 1-8, Supplementary Table 1, Supplementary Methods and Supplementary References [file ncomms10586-s1.pdf]

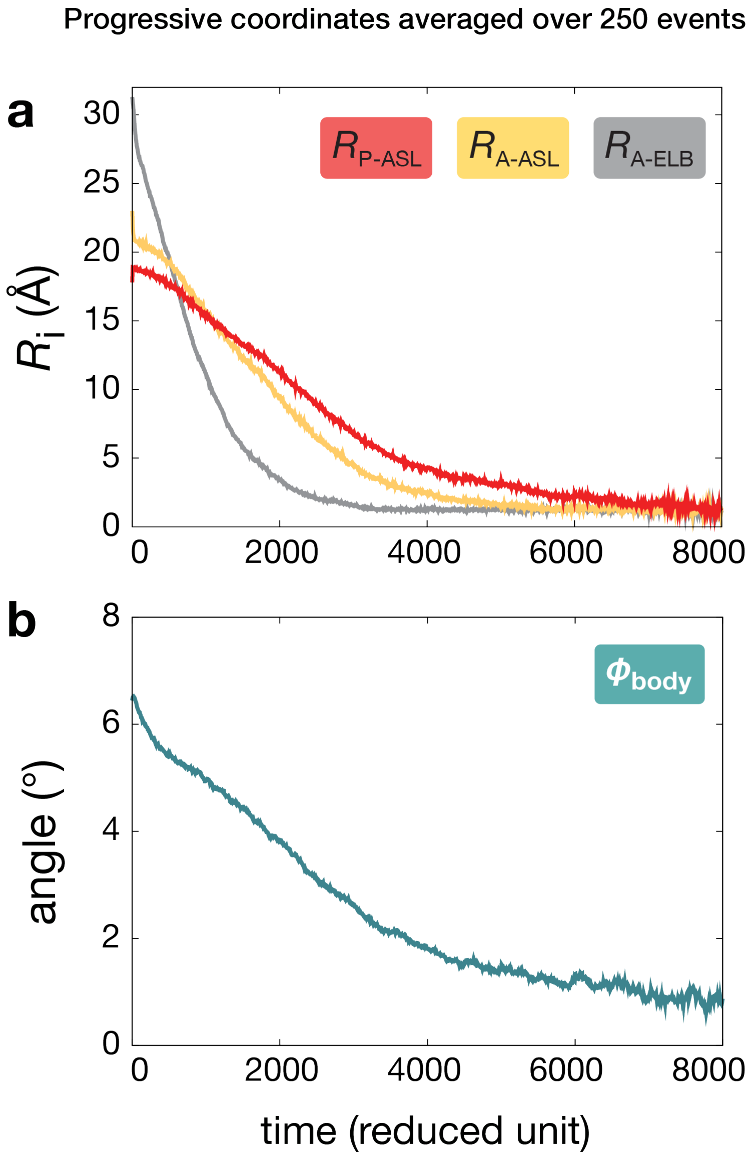

SUPPLEMENTARY FIG. 1. **(a)** tRNA coordinates (as shown in Fig. 2c of the main text) averaged over 250 trajectories. These average time traces implicate the same order of tRNA movements as observed for individual simulations (cf. Fig. 2c of the main text). Specifically, the elbow of the A-site tRNA first moves on the large subunit into the P site ($R_{\text{A-ELB}} \rightarrow 0$; grey line). Then the ASLs of the tRNAs move on the small subunit into the P and E sites ($R_{\text{A-ASL}} \rightarrow 0$ and $R_{\text{P-ASL}} \rightarrow 0$; yellow and red lines), where the A-ASL reaches its endpoint before the P-ASL. **(b)** Average time trace of body rotation (ϕ_{body}) over 250 runs shows a monotonic decrease of intersubunit rotation during the transition between the initial A/A-P/E and final P/P-E/E configurations. Note that the average time traces for head rotation and head tilting are not included, since these head coordinates are not progress coordinates of the 30S translocation process. That is, the head rotation and tilting angles are not monotonically related to translocation and they can not distinguish between the endpoints. Since head motions occur at different times for different simulations, averaging masks the large-scale fluctuations of the head that occur in individual trajectories.

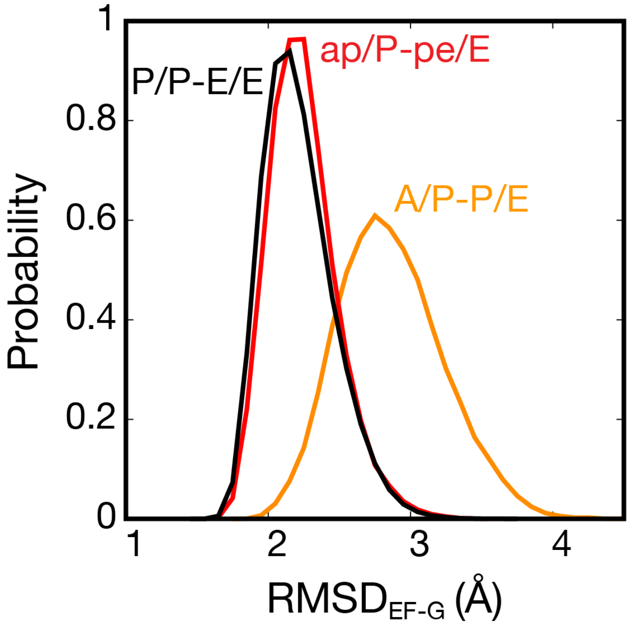

SUPPLEMENTARY FIG. 2. Probability distribution as a function of the RMSD of EF-G relative to the post-bound conformation ($RMSD_{EF\text{-}G}$), calculated for the A/P-P/E, ap/P-pe/E, and P/P-E/E ensembles. The distribution for the ap/P-pe/E ensemble (red curve) is virtually identical to that of the P/P-E/E ensemble (black curve). This suggests that when the ribosome adopts the chimeric ap/P-pe/E configuration, EF-G is in a post-translocation–like conformation, where its domain IV is extended towards the A site of the 30S subunit.

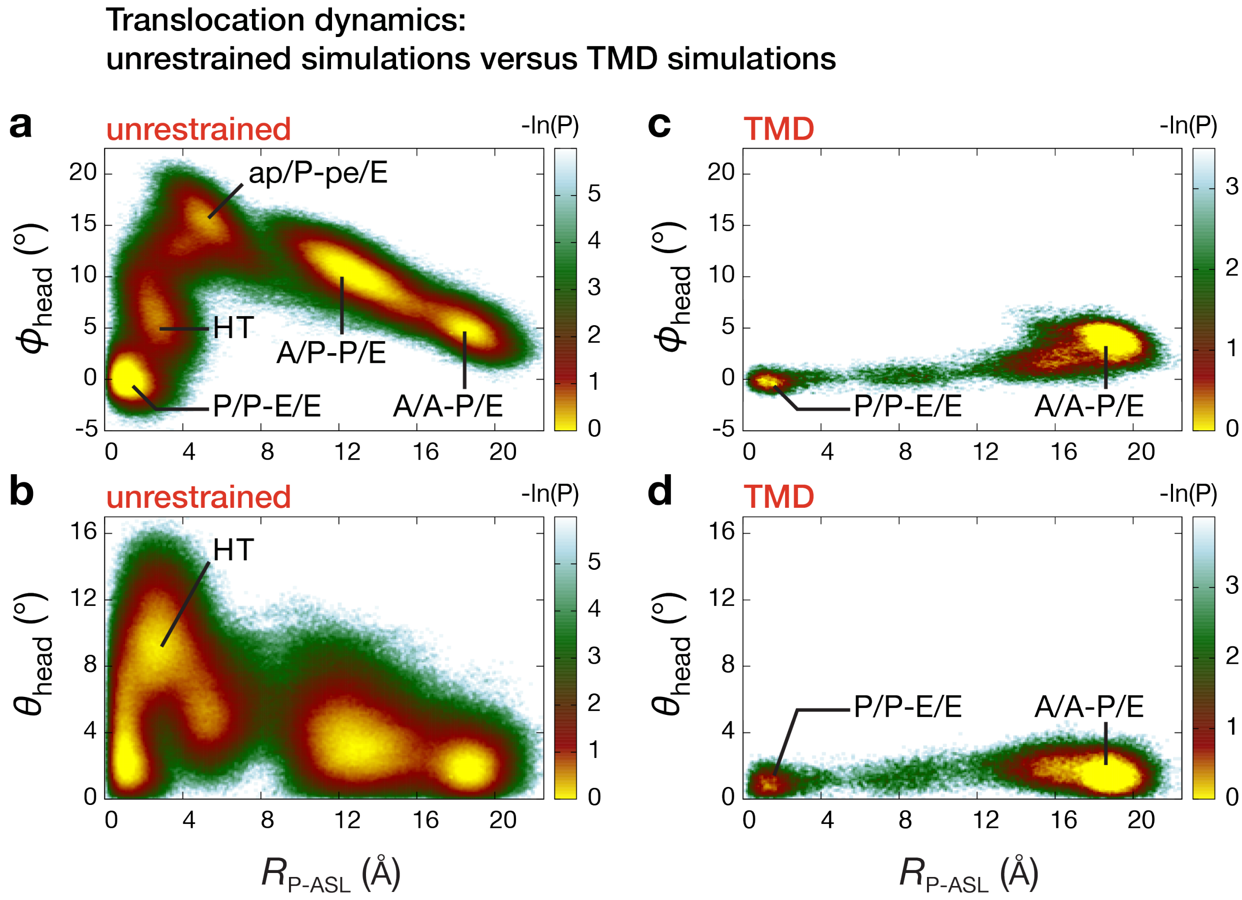

SUPPLEMENTARY FIG. 3. Probability distributions **(a)** $P(R_{\text{P-ASL}}, \phi_{\text{head}})$ and **(b)** $P(R_{\text{P-ASL}}, \theta_{\text{head}})$ calculated from unrestrained simulations. These data are the same as presented in Fig. 3a,b of the main text, but shown from the A/A-P/E ensemble. In the main text, where translocation on the 30S subunit was the focus, movement from the A/A-P/E to the A/P-P/E ensemble was considered as initial equilibration period and was not included in the analysis. To compare the unrestrained dynamics with targeted translocation events, we performed 100 TMD simulations. From these targeted simulations, the probability distributions **(c)** $P(R_{\text{P-ASL}}, \phi_{\text{head}})$ and **(d)** $P(R_{\text{P-ASL}}, \theta_{\text{head}})$ were also calculated, analogous to Panels **a** and **b**. These TMD results clearly show that even weak targeting forces can have a dramatic impact on the dynamics, relative to unrestrained simulations. Here, the presence of a TMD potential precludes spontaneous large-scale fluctuations (e.g. large-scale rotation of the head), and prevents the formation of intermediate state ensembles.

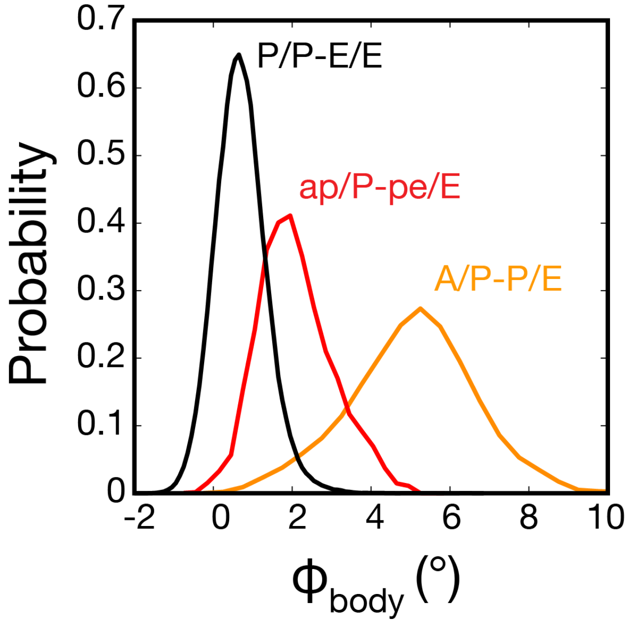

SUPPLEMENTARY FIG. 4. Probability as a function of body rotation (ϕ_{body}) calculated for the A/P-P/E, ap/P-pe/E, and P/P-E/E ensembles. Peak values of ϕ_{body} show that the average body rotation relaxes from $\sim 6°$ to $\sim 2°$ to $\sim 0°$ during the transition between the A/P-P/E, ap/P-pe/E, and P/P-E/E ensembles. While the range of values for each ensemble may appear large, the distribution of values is consistent with the range of values observed in a $1.3\,\mu$s explicit-solvent simulation of an A/A-P/P ribosome [1].

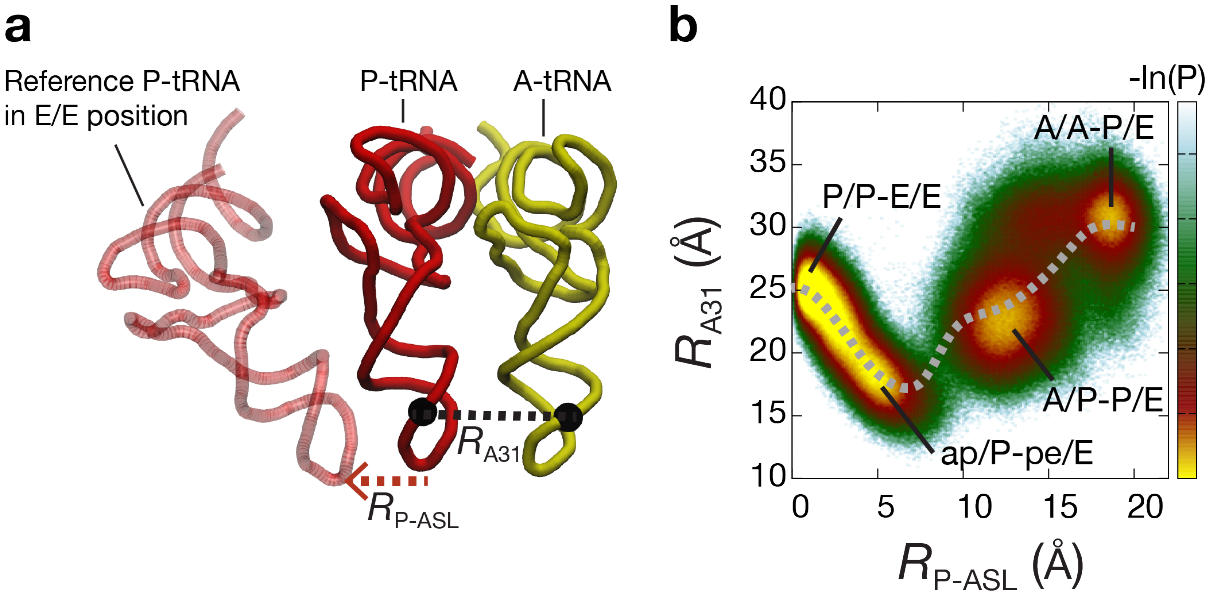

SUPPLEMENTARY FIG. 5. **(a)** To describe compaction between tRNA ASLs during translocation, we calculated the distance between the P atoms of A31 in the tRNAs: R_{A31}. As introduced earlier, $R_{\text{P-ASL}}$ represents the distance from the ASL of the P-tRNA to the E site of the 30S body. **(b)** $P(R_{\text{P-ASL}}, R_{\mathrm{A31}})$ together with $\bar{R}_{\mathrm{A31}}(R_{\text{P-ASL}})$ (average compaction as a function of P-tRNA ASL position, shown by dashed line) reveal a large degree of ASL compaction in the ap/P-pe/E ensemble ($\bar{R}_{\mathrm{A31}} \approx 18$–$20$ Å). In contrast, in the A/A-P/E and P/P-E/E ensembles (i.e. the endpoints of the simulations) the average compaction is $\sim$30 Å and $\sim$25 Å.

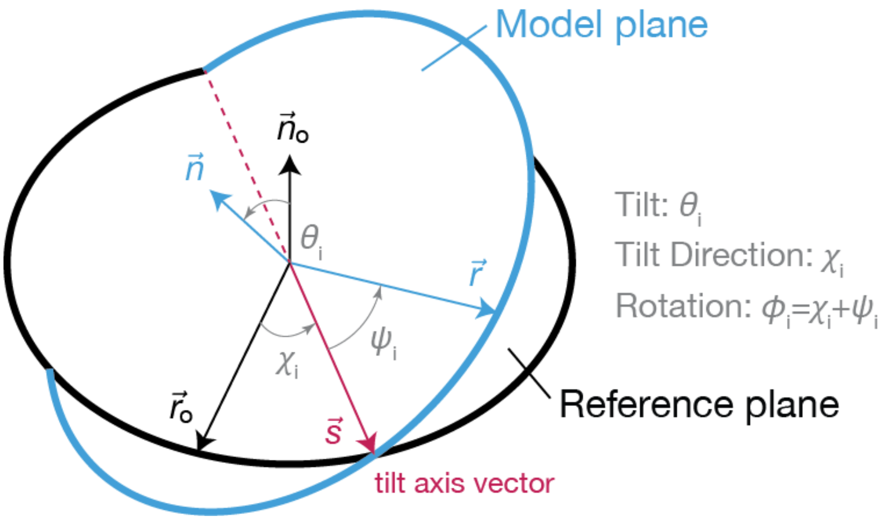

SUPPLEMENTARY FIG. 6. **Rotation coordinates for the 30S head and 30S body.** The same steps were followed to calculate the rotation coordinates for the 30S head and 30S body. The subscript "i" of the angle variables stands for either "head" or "body". Rotation angles are determined by comparing the reference vectors $\vec{r}$ and $\vec{n}$ of the model plane (blue) with the corresponding vectors $\vec{r}_o$ and $\vec{n}_o$ of the reference plane (black). Tilting occurs about the vector $\vec{s} = \vec{n}_o \times \vec{n}$ (red) according to the right-hand rule. The magnitude of tilt is θ_{i}. The direction of tilt (i.e. the orientation of vector $\vec{s}$) is described by χ_{i}. Counterclockwise rotations along χ_{i} and ψ_{i} are defined as positive angles. 30S head/body rotation is defined as $\phi_{\mathrm{i}} = \chi_{\mathrm{i}} + \psi_{\mathrm{i}}$.

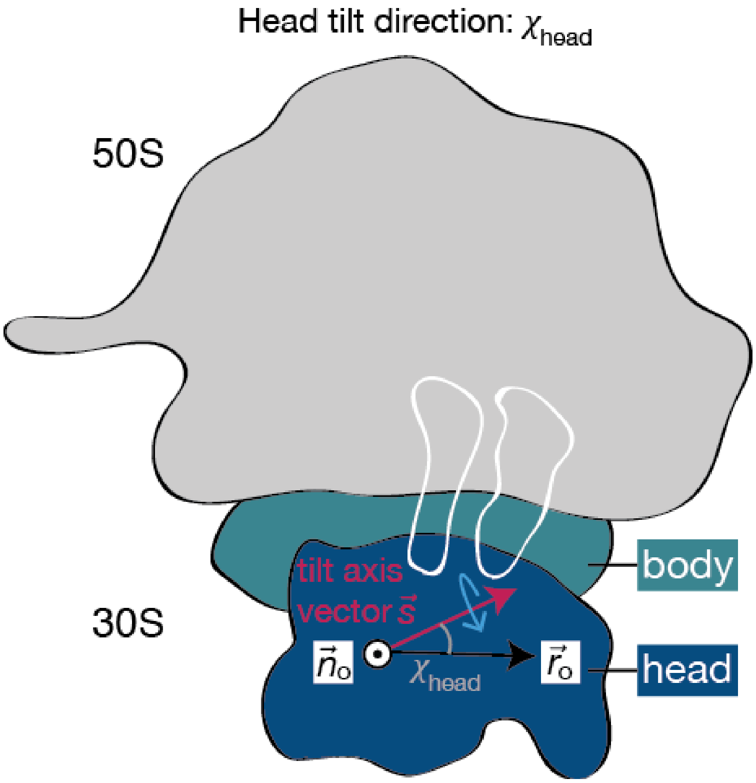

SUPPLEMENTARY FIG. 7. Schematic representation of the top view of the $\vec{n}_o$-plane of the 30S head shown in Supplementary Fig. 6. The reference vector $\vec{r}_o$ (black) was defined such that it is parallel to the axis of the mRNA. Head tilt direction is described by χ_{head}, which is the angle between the reference $\vec{r}_o$ and the tilt axis vector $\vec{s}$ (red). Tilting about $\vec{s}$ (indicated by blue arrow) follows the right-hand rule (cf. Supplementary Fig. 6).

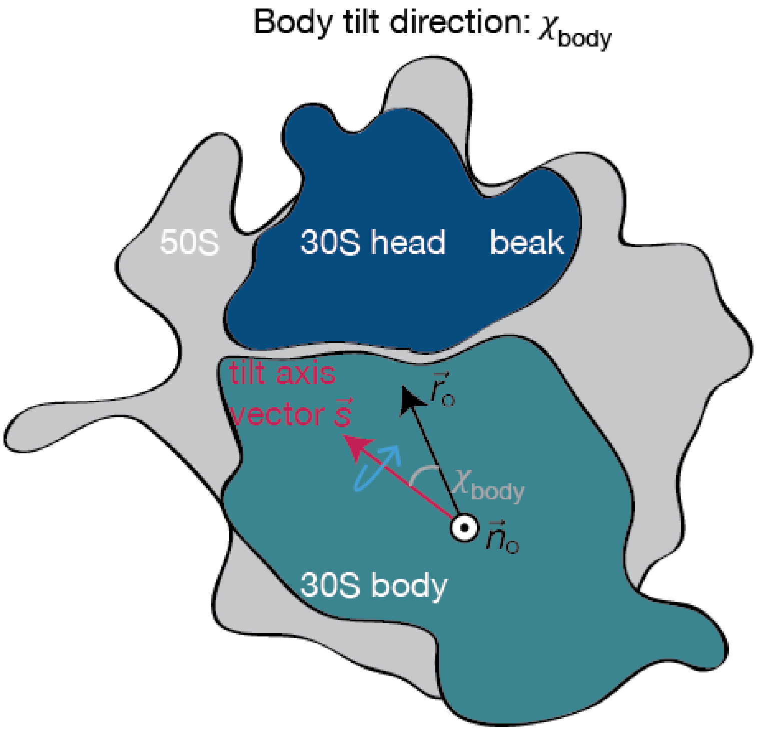

SUPPLEMENTARY FIG. 8. Schematic representation of the top view of the $\vec{n}_o$-plane of the 30S body shown in Supplementary Fig. 6. The reference vector $\vec{r}_o$ (black) was defined such that it aligns with the projection of h44 onto the $\vec{n}_o-$plane. The direction of body tilt is described by χ_{head}, which is the angle between the reference $\vec{r}_o$ and the tilt axis vector $\vec{s}$ (red). Tilting about vector $\vec{s}$ (indicated by blue arrow) follows the right-hand rule (cf. Supplementary Fig. 6).

SUPPLEMENTARY TABLE 1. Comparison of the simulated ap/P-pe/E ensemble with experimental data

References	PDB	tRNA	ϕ_{head} (°)	θ_{head} (°)	χ_{head} (°)	ϕ_{body} (°)	R_{gate} (Å)	R_{pe} (Å)	R_{A31} (Å)
Simulated ap/P-pe/E ens.	—	pe/E, ap/P	16.5±1.5	5.2 ± 1.7	1.7±13.2	1.9 ± 1.2	27.7±1.1	11.5±1.3	19.1±1.6
Ramrath et al. (2013) [2]	3J5N	pe/E, ap/P	17.8	3.1	11.7	3.2	25.1	12.2	21.3
Zhou et al. (2014) [3]	4QS0	pe/E, ap/ap	20.1	3.2	59.4	3.0	26.2	12.4	22.5
Zhou et al. (2013) [4]	4KCY	pe/E	16.6	2.6	51.5	1.6	24.3	11.9	—
Ratje et al. (2010) [5]	2XUY	pe/E	19.1	3.1	34.3	3.2	26.8	10.6	—

SUPPLEMENTARY METHODS

Preparation of the classical A/A-P/P and P/P-E/E structures used for the construction of the structure-based forcefield

Atomic coordinates of the *T. Thermophilus* 70S ribosome with three $tRNA^{Phe}$ molecules were taken from Jenner et al. [6] (PDB ID code 4V6F). This structure lacks protein L1 and elongation factor G (EF-G). Based on this structure, classical A/A-P/P and P/P-E/E configurations with the L1 protein and EF-G were prepared, as described in Ref. [7]. To alleviate any atomic conflicts, these two classical configurations were then refined by energy minimization in explicit solvent. Energy minimization was performed with the Gromacs (v4.6.1) software package [8, 9], using the AMBER99 forcefield [10] and SPC/E [11] water model. Each configuration was solvated in a triclinic box with a 10 Å buffer on all sides. 100 mM of K^+ ions were added to the system. Cl^- ions were then added such that the net charge of the system was neutral. Energy minimization was conducted in multiple stages, and each stage was carried out using the steepest descent algorithm: 1) Unrestrained energy minimization was run for $50{,}000$ steps. This step alleviated unfavorable interactions within the ribosome and allowed solvent and ions to relax. 2) Energy minimization was performed using harmonic position restraints on backbone atoms with respect to the initial configuration. Harmonic restraints were applied with a force constant of 1 kJ mol^{-1} Å^{-2}, and energy minimization was run for $20{,}000$ steps. 3) Restrained energy minimization as described for step 2 was performed with an increased restraint force constant of 100 kJ mol^{-1} Å^{-2}. During the restrained energy minimization stages 2 and 3, the solvent and ions were allowed to further relax without affecting the backbone structure of the initial configuration. Steps 1, 2, and 3 were repeated before performing final unrestrained energy minimization.

Preparation of the atomic structures used as initial configurations in the simulations

The A/A-P/E and ap/P-pe/E configurations with protein L1 and EF-G were prepared according to Whitford et al. [7]. In Ref. [7], the A/A-P/E and ap/P-pe/E configurations are referred to as TI^{pre} and TI^{post}. These configurations were also refined by energy minimization in explicit solvent, using the same protocol as described above.

The refined A/A-P/E structure was used as the initial configuration for the full translocation

simulations (Figs. 2–4 of the main text). The refined ap/P-pe/E structure was used as the initial configuration for the simulations with the modified forcefields where sterics of the PE-loop and/or protein S13 were removed (Fig. 5 of the main text).

Details of the forcefield

Structure-based (SMOG) model

To simulate translocation, we used an all-atom multi-basin structure-based (SMOG) model [12]. All heavy (non-hydrogen) atoms (154,723) are explicitly represented as spherical beads of unit mass. In a single-basin SMOG model, an experimentally-derived structure is defined as the potential energy minimum, and the functional form of the potential is given by:

$$\begin{aligned} V = &\sum_{bonds} \epsilon_r (r - r_o)^2 + \sum_{angles} \epsilon_\theta (\theta - \theta_o)^2 + \sum_{impropers} \epsilon_\chi (\chi - \chi_o)^2 \\ &+ \sum_{backbone\ dihedrals} \epsilon_{bb}\, F(\phi) + \sum_{sidechain\ dihedrals} \epsilon_{sc}\, F(\phi) \\ &+ \sum_{contacts} \epsilon_c \left[\left(\frac{\sigma_{ij}}{r_{ij}} \right)^{12} - 2 \left(\frac{\sigma_{ij}}{r_{ij}} \right)^{6} \right] + \sum_{non-contacts} \epsilon_{nc} \left(\frac{\sigma_{nc}}{r_{ij}} \right)^{12} \end{aligned} \quad (1)$$

where $F(\phi) = [1 - \cos(\phi - \phi_o)] + \frac{1}{2}[1 - \cos(3(\phi - \phi_o))]$, and $\{r_o\}$, $\{\theta_o\}$, $\{\chi_o\}$, $\{\phi_o\}$, and $\{\sigma_{ij}\}$ are the values found in the input structure. The energy scale is set to $\epsilon = 1$, and the coefficients are given values $\epsilon_r = 50\epsilon/\text{Å}^2$, $\epsilon_\theta = 40\epsilon/\text{rad}^2$, $\epsilon_\chi = 40\epsilon/\text{rad}^2$, $\epsilon_{nc} = 0.1\epsilon$, and $\sigma_{nc} = 2.5\text{Å}$. The dihedral and contact energies were weighted such that

$$\frac{\epsilon_{bb}}{\epsilon_{sc}} = 2 \text{ for proteins (1 for RNA)},$$

$$\frac{\sum \epsilon_c}{\sum \epsilon_{bb} + \sum \epsilon_{sc}} = 2 \text{ and}$$

$$\sum \epsilon_c + \sum \epsilon_{bb} + \sum \epsilon_{sc} = N\epsilon,$$

where N is the number of atoms. Contact interactions were described by the 6-12 potential, and contacts were assigned based on the Shadow Contact Map algorithm [13].

Construction of the Multi-basin SMOG model

Single-basin models based on the A/A-P/P and P/P-E/E configurations were generated using the smog-server webtool (http://smog-server.org) [14]. All stabilizing interactions that are found in the P/P-E/E model (which includes EF-G) were included. Additionally, intermolecular contacts between the ribosome and mRNA-tRNA formed in the A/A-P/P model were added as stabilizing interactions. The strength of the contacts between the ribosome and mRNA-tRNA were rescaled by 0.3, to account for transient nature of mRNA-tRNA binding, relative to the lifetime of the ribosome. This construction defines the A/P-P/P and P/P-E/E configurations as dominant minima on the potential energy surface, and allows mRNA-tRNA to move between binding sites.

Interface contacts between the 30S and 50S subunits, and between the 30S head and body were weakened, to account for the fact that intra- and intersubunit rotations continuously occur in solution. With respect to the 30S-50S interface, the center of the interface was defined as the geometric center of the atoms involved in intersubunit contacts. The strength of intersubunit contacts within a radius of $20\,\text{Å}$ from the center was then rescaled by a factor of 0.3. And the strength of intersubunit contacts outside the radius was rescaled by 0.1. Defining weaker contacts on the outside is consistent with biochemical probing data suggesting that intersubunit bridges at the periphery of the interface are more flexible than bridges that are closer to the center [15]. Finally, to account for head movements, the strength of the intrasubunit contacts between the head and body were rescaled by 0.3. Here, the head domain is defined as residues C930 to C1388 in the 16S rRNA and proteins S3, S7, S9, S10, S13, S14, and S19.

Since EF-G transiently associates with the ribosome during the elongation cycle, the strength of the contacts between EF-G and the ribosome was rescaled. In preliminary simulations, the scaling factor was given values of 0.1, 0.2, 0.3, $\ldots$, or 1.0. For production simulations, a factor of 0.6 was used since it was found to be sufficiently strong that EF-G would not prematurely dissociate from the ribosome during translocation. Further, all stabilizing interactions between domain IV of EF-G and ribosome-mRNA-tRNA were removed, so that domain IV only sterically interacts with the assembly.

It is instructive to note that the relative stability of the A/A-P/P and P/P-E/E conformations is determined by the differences in the number of attractive tRNA interactions with the A, P and E sites, as well as the presence of EF-G. In this model, the stabilizing energy is directly proportional

to the number of tRNA-ribosome contacts found in the A/A-P/P and P/P-E/E crystal structures. Due to the presence of interactions between the E/E-tRNA and the L1 stalk, there are more tRNA-ribosome contacts when a tRNA is in the E/E conformation, than in the A/A conformation. This leads to a net difference of approximately 10 units of energy (reduced units) between the A/A-P/P and P/P-E/E conformations. This difference in stability is relatively small compared to the scale of fluctuation in the potential energy of each tRNA, which is approximately 20 units of energy. Thus, the character of the dynamics is largely determined by thermal fluctuations, which is consistent with the notion of the ribosome acting as a Brownian ratchet machine. The second factor that can affect the relative stability of the endpoints is the presence of EF-G. As shown in Supplementary Fig. 2, EF-G adopts a post-translocation–like conformation in the ap/P-pe/E ensemble (as also seen in cryo-EM reconstructions and crystal structures [2–5]). Hence, EF-G can influcence the relative stability towards the P/P-E/E ensemble by preventing tRNA reverse movement.

Variations of the multi-basin SMOG model

The following variations to the multi-basin model were introduced, to test the relative role of protein S13 and the PE-loop (G1338-U1341) during translocation (data shown in Fig. 5 of the main text): Model 1) Same model as described above; Model 2) Identical to model 1, except excluded-volume (i.e. steric) interactions between S13 and the A-tRNA were not included; Model 3) Identical to model 1, except steric interactions between the PE-loop and the P-tRNA were not included; and Model 4) Identical to model 1, except steric interactions between both S13, PE-loop and tRNA were not included.

To exclude steric interactions between S13/PE-loop and tRNA, the repulsive $\frac{1}{r^{12}}$ non-contact terms in Eq. (1) were not included between S13 and A-tRNA (Model 2), between PE-loop and P-tRNA (Model 3), or between S13, PE-loop and tRNA (Model 4).

Targeted molecular dynamics (TMD) description: Harmonic implementation

All translocation data presented in the main text were obtained from unrestrained simulations. To compare and contrast those spontaneous, unrestrained translocation events with restrained, targeted dynamics, we performed 100 TMD simulations of the transition from the A/A-P/E to the P/P-E/E configuration (see results in Supplementary Fig. 3). That is, in addition to the multi-basin

structure-based model described above (referred to as Model 1), which provides an underlying energy landscape, TMD simulations include an additional time-dependent potential that biases the simulation towards the endpoint P/P-E/E configuration. Here, the TMD potential is of the harmonic form

$$V_{\text{TMD}}(t) = \epsilon_{\text{TMD}} \left[\text{RMSD}(t) - D(t)\right]^2, \tag{2}$$

where RMSD(t) is the weighted root-mean-square deviation,

$$\text{RMSD}(t) = \sqrt{\frac{1}{N}\sum_{k=1}^{N} w_k \left[\vec{r}_k(t) - \vec{r}_{k,\,\text{target}}\right]^2}. \tag{3}$$

In the equation above, N is the number of atoms, $\vec{r}_k(t)$ denotes the position of atom k at time t, and $\vec{r}_{k,\,\text{target}}$ represents the target location of that atom. The values of w_k were set such that $w_k = 1$ if the distance between the initial and the final (target) position of the k-atom, $R_k^{\text{f–i}}$, is below the threshold $R_{\text{thr}} = 20\,\text{Å}$, and $w_k = \frac{R_{\text{thr}}}{R_k^{\text{f–i}}}$ if $R_k^{\text{f–i}} > R_{\text{thr}}$. Note that the force on individual atoms is proportional to the distance from the target. By introducing a threshold distance, above which the weights are reduced, we ensure that atoms undergoing larger rearrangements will not be subject to much larger forces. Finally, $D(t)$ is a linear function in time, which descreases from RMSD$_{\text{initial}}$ (i.e. RMSD between the A/A-P/E and P/P-E/E configurations) to zero, over a prescribed time interval τ, and remains zero for the rest of the simulation. That is, $D(t)$ is given by

$$D(t) = \begin{cases} \text{RMSD}_{\text{initial}}\left(1 - \frac{t}{\tau}\right), & t \leq \tau \\ 0, & t > \tau \end{cases} \tag{4}$$

TMD simulation details

Each TMD simulation was initiated at the A/A-P/E configuration, where the coordinates of the P/P-E/E configuration were used as the target. All atoms (154,723) were included in the RMSD calculation, and RMSD$_{\text{initial}} = 6.6\,\text{Å}$. Reduced units were used for all calculations. Each simulation was performed for 3.5×10^5 time steps of size 0.002. The effective timescale of each simulation may be estimated as microseconds. See Ref. [16] and the supplementary material of Ref. [17] for detailed discussion on timescale estimates in SMOG models. The same reduced temperature of 0.5 was used as for the unrestrained simulations described in the main text. $D(t)$ was linearly reduced

in time according to Eq. (4) for 2.5×10^5 time steps. The energetic weight ϵ_{TMD} of the TMD potential in Eq. (2) was set to 2000ϵ (where $\epsilon = 1$). Note that the value of ϵ_{TMD} used here is only 10% of that in previous TMD simulations of tRNA hybrid state formation [18].

Details of the reaction coordinates

VMD scripts that calculate all rotation angles are available on the Whitford Research Group webpage.

Reaction coordinates for the tRNAs

Three coordinates were defined to measure the movement of the P- and A-site tRNAs. $R_{\text{P-ASL}}$ and $R_{\text{A-ASL}}$ describe the movement of the ASLs of the P- and A-site tRNAs relative to the 30S body. They are the distances of the tRNAs to their positions when in the E/E and P/P sites. Similarly, $R_{\text{A-ELB}}$ describes the movement of the elbow of the A-site tRNA relative to the 50S subunit, or the distance of the A-tRNA elbow to its location when in the P/P position.

To calculate the elbow coordinate $R_{\text{A-ELB}}$ for a given trajectory frame, the 23S of the simulated structure was aligned to the reference P/P-E/E structure. $R_{\text{A-ELB}}$ was then defined as the distance of the U60:P atom in the A-tRNA to its location in the reference P/P-E/E structure.

The ASL coordinates $R_{\text{P-ASL}}$ and $R_{\text{A-ASL}}$ were determined after 16S-body alignment of the simulated structure to the P/P-E/E structure. $R_{\text{P-ASL}}$ (or $R_{\text{A-ASL}}$) was then defined as the distance of the A36:P atom in the P-tRNA (or A-tRNA) to its location in the reference P/P-E/E structure.

The 23S and 16S-body alignments were performed using the core residues in the respective domains, as described elsewhere [1].

Reaction coordinates for the 30S head

To describe the motions of the 30S head, we first defined a reference plane that represents the classical orientation of the head (Supplementary Fig. 6). This plane is fixed by three atoms within the head of the classical A/A-P/P configuration, which are A977:P, C1298:O2P, and A1374:P. Using these atoms, vectors between atom pairs were set as $\vec{r}_o = position[\text{A977:P}] - position[\text{A1374:P}]$ and $\vec{x}_o = position[\text{C1298:O2P}] - position[\text{A1374:P}]$. The normal vector of the reference plane can

then be written as $\vec{n}_o = \vec{r}_o \times \vec{x}_o$. The atom-pair vector $\vec{r}_o$ and the normal $\vec{n}_o = \vec{r}_o \times \vec{x}_o$ were used as reference vectors. Note that the three atoms mentioned above were chosen such that the resulting reference plane (fixed by $\vec{n}_o$) is consistent with the plane defined for head rotation in Ref. [1].

To calculate rotation angles of the head for a given structure, we employ the following protocol, which is an extension of the method reported in Ref. [1]:

1. The core residues of the 16S body are used to align the structural model to the reference A/A-P/P structure.

2. The core residues of the 16S head are used to align the head of the A/A-P/P reference to the rotated head of the model structure.

3. From step 2) we obtain the reference vectors of the aligned (i.e. rotated) reference head of the A/A-P/P structure, which are denoted by $\vec{r}$ and $\vec{n} = \vec{r} \times \vec{x}$. These reference vectors of the rotated head-plane are compared with $\vec{r}_o$ and $\vec{n}_o = \vec{r}_o \times \vec{x}_o$ of the unrotated reference head-plane (Supplementary Fig. 6).

4. Calculate the tilt axis vector $\vec{s}$ that defines the line of nodes of the two planes: $\vec{s} = \vec{n}_o \times \vec{n}$.

5. Calculate rotation angle ϕ_{head}: head rotation is defined as $\phi_{\text{head}} = \chi_{\text{head}} + \psi_{\text{head}}$. As shown in Supplementary Fig. 6, χ_{head} is the angle between $\vec{r}_o$ and $\vec{s}$, and ψ_{head} is the angle between $\vec{s}$ and $\vec{r}$. Counterclockwise rotations along χ_{head} and ψ_{head} are defined as positive angles.

6. Calculate tilt angles: the magnitude of head tilt is denoted by θ_{head} which is simply the angle between $\vec{n}_o$ and $\vec{n}$. Tilting occurs about the axis vector $\vec{s}$ and follows the right-hand rule. The orientation of $\vec{s}$, which is the direction of head tilt, is described by the angle χ_{head}.

Note that the alignment performed in step 2) provides an average orientation of the head, or “idealized” coordinates, as described in Ref. [1]. Using the idealized coordinates of the aligned (i.e. rotated) reference head structure, we ensure that collective movement of the 30S head is measured, and not individual atomic fluctuations.

To define a reference direction corresponding to the tilt direction angle $\chi_{\text{head}} = 0$, the reference vector $\vec{r}_o$ was rotated in the $\vec{n}_o-$plane by a constant angle $\alpha = -35^\circ$ (clockwise rotation as viewed from the top of the head) such that $\vec{r}_o$ points towards the 30S A site and aligns with the axis of the mRNA between the A- and P-site codons (axis between the P atoms of U48 and U45 in the

mRNA in the A/A-P/P structure). With these definitions, $\chi_{\text{head}} = 0$ corresponds to tilting around the mRNA axis, where the head moves away from the 30S-50S interface (see Supplementary Fig. 7 for a schematic illustration of head tilt direction).

In simulation, the model structure, for which rotation angles are calculated, corresponds to a given frame of the trajectory. We used the *trjconv* module of Gromacs to align each simulated frame to the reference (same reference as used in step 1), based on the 16S-body-core residues. Next, the coordinates of the 30S head were idealized through 16S-head-core alignment (step 2). Using the idealized coordinates of each frame, the vectors and angles were calculated for each frame with Gromacs modules and in-house scripts.

Previously in Ref. [1], head rotation was calculated by projecting the rotated reference vector $\vec{r}$ onto the reference $\vec{n}_o$-plane. Let this projection be $\vec{r}'$, then head rotation was defined as the angle between $\vec{r}_o$ and $\vec{r}'$. That protocol can lead to false detection of rotation angles when tilting is present. Consider, for example, the case where only tilting occurs and no rotation is present. Further, let this tilting be about an axis vector $\vec{s}$ that does not align with the reference $\vec{r}_o$ (i.e. when $\chi_{\text{head}} \neq 0$). In such case, the projection $\vec{r}'$ and the reference $\vec{r}_o$ can form nonzero rotation angles, even in the absence of any rotation. In the tilt-only case, it is $\chi_{\text{head}} = -\psi_{\text{head}}$, and according to our rotation definition here: $\phi_{\text{head}} = \chi_{\text{head}} + \psi_{\text{head}} = 0$ (step 5). That is, using an Euler-angle based set of coordinates eliminates the ocurrance of artificial rotation angle values. As a point of comparison with other coordinate definitions, it is worth noting that when there is no tilting, ϕ_{head} will yield identical values to the Euler-Rodrigues angle employed by Mohan, Donohue and Noller [19]. While the values of the two coordinates are quantitatively similar, we chose to use Euler angles directly as they allow for tilting to be separately described.

Reaction coordinates for the 30S body

The rotation coordinates for the 30S body: ϕ_{body}, θ_{body}, and χ_{body} were defined and calculated following the same strategy as for the 30S head. For completeness we will provide all the details explicitly. In this study, with respect to the motions of the body, we observe a signal for body rotation (Supplementary Fig. 4) during translocation, but not for body tilting (also known as "subunit rolling" in the context of the 80S ribosome [20]). That is, here, the average tilt angle of the body ($\bar{\theta}_{\text{body}}$) is only $1.4°$.

To calculate the body rotation angles, a plane that represents the classical orientation of the body was defined (Supplementary Fig. 6). This reference plane is fixed by three atoms within the body of the classical A/A-P/P structure. These atoms are C237:P, C811:P, and G1488:O6. Using the same vector notation as for the head case above, vectors between atom pairs were set as $\vec{r}_o = position$[G1488:O6]$-position$[C237:P] and $\vec{x}_o = position$[C811:P]$-position$[C237:P]. The normal vector of the reference plane can be written as $\vec{n}_o = \vec{r}_o \times \vec{x}_o$. Then $\vec{r}_o$ and $\vec{n}_o$ were used as reference vectors. Note that the three atoms in the body were chosen such that the resulting $\vec{n}_o$-plane is consistent with the plane defined elsewhere for body rotation [1].

To calculate rotation angles of the body for a given model structure, we employed the following protocol:

1. The core residues of the 23S are used to align the model structure to the 23S of the reference A/A-P/P structure.

2. The core residues of the 16S body are used to align the body of the A/A-P/P reference to the rotated body of the model structure.

3. From step 2 we obtain the reference vectors of the aligned (i.e. rotated) reference body of the A/A-P/P structure, which are denoted by $\vec{r}$ and $\vec{n} = \vec{r} \times \vec{x}$. These reference vectors of the rotated body-plane will be compared with $\vec{r}_o$ and $\vec{n}_o = \vec{r}_o \times \vec{x}_o$ of the unrotated reference body-plane (Supplementary Fig. 6).

4. Calculate the vector $\vec{s}$ that defines the line of nodes of the two planes: $\vec{s} = \vec{n}_o \times \vec{n}$.

5. Calculate rotation angle ϕ_{body}: body rotation is defined as $\phi_{\text{body}} = \chi_{\text{body}} + \psi_{\text{body}}$. As shown in Supplementary Fig. 6, χ_{body} is the angle between $\vec{r}_o$ and $\vec{s}$, and ψ_{body} is the angle between $\vec{s}$ and $\vec{r}$. Counterclockwise rotations along χ_{body} and ψ_{body} are defined as positive angles.

6. Calculate tilt angles: the magnitude of body tilt is denoted by θ_{body} which is simply the angle between $\vec{n}_o$ and $\vec{n}$. Tilting occurs about the axis vector $\vec{s}$ and follows the right-hand rule. The orientation of $\vec{s}$, which is the direction of body tilt, is described by the angle χ_{body}.

Note that the alignment in step 2) provides an average orientation of the body (i.e. "idealized" coordinates, as introduced in Ref. [1]). Using these idealized coordinates of the aligned (i.e.

rotated) reference body structure, we ensure that collective movement of the body domain is measured, and not individual atomic fluctuations.

As explained above for 30S head rotation, the rotation definition in step 5) ensures that tilt movements do not lead to false detection of rotations.

The reference vector $\vec{r}_o$ was chosen, such that it points towards the head and aligns with the axis of h44. That is, $\chi_{\text{body}} = 0$ corresponds to tilting roughly around the h44 axis, where the beak of the 30S is displaced towards the 30S-50S interface (see Supplementary Fig. 8 for a schematic illustration of head tilt direction).

In simulation, the model structure, for which rotation angles are calculated, corresponds to a given frame of the trajectory. We used the *trjconv* module of Gromacs to align each simulated frame to the A/A-P/P reference, based on the 23S-core residues (step 1). Next, the coordinates of the 30S body were idealized through 16S-body-core alignment (step 2). Using the idealized coordinates of each frame, the vectors and angles were calculated for each frame.

Supplementary References

[1] Whitford, P. C., Blanchard, S. C., Cate, J. H. D. & Sanbonmatsu, K. Y. Connecting the kinetics and energy landscape of tRNA translocation on the ribosome. *PLoS Comput. Biol.* **9**, e1003003 (2013).

[2] Ramrath, D. J. F. *et al.* Visualization of two transfer RNAs trapped in transit during elongation factor G-mediated translocation. *Proc. Natl. Acad. Sci. USA* **110**, 20964–9 (2013).

[3] Zhou, J., Lancaster, L., Donohue, J. P. & Noller, H. F. How the ribosome hands the A-site tRNA to the P site during EF-G-catalyzed translocation. *Science* **345**, 1188–1191 (2014).

[4] Zhou, J., Lancaster, L., Donohue, J. P. & Noller, H. F. Crystal structures of EF-G-ribosome complexes trapped in intermediate states of translocation. *Science (New York, N.Y.)* **340**, 1236086 (2013).

[5] Ratje, A. H. *et al.* Head swivel on the ribosome facilitates translocation by means of intra-subunit tRNA hybrid sites. *Nature* **468**, 713–716 (2010).

[6] Jenner, L. B., Demeshkina, N., Yusupova, G. & Yusupov, M. Structural aspects of messenger RNA reading frame maintenance by the ribosome. *Nat. Struct. Mol. Biol.* **17**, 555–60 (2010).

[7] Whitford, P. C. *et al.* Excited states of ribosome translocation revealed through integrative molecular modeling. *Proc. Natl. Acad. Sci. USA* **108**, 18943–18948 (2011).

[8] Lindahl, E., Hess, B. & van der Spoel, D. Gromacs 3.0: A package for molecular simulation and trajectory analysis. *J. Mol. Model.* **7**, 306317 (2001).

[9] Hess, B., Kutzner, C., van der Spoel, D. & Lindahl, E. GROMACS 4: Algorithms for highly efficient, load-balanced, and scalable molecular simulation. *J. Chem. Theory Comput.* **4**, 435–447 (2008).

[10] Wang, J. M., Cieplak, P. & Kollman, P. A. How well does a restrained electrostatic potential (RESP) model perform in calculating conformational energies of organic and biological molecules? *J. Comput. Chem.* **21**, 1049–1074 (2000).

[11] Berendsen, H. J. C., Grigera, J. R. & Straatsma, T. P. The missing term in effective pair potentials. *J. Phys. Chem.* **91**, 6269–6271 (1987).

[12] Whitford, P. C. *et al.* An all-atom structure-based potential for proteins: Bridging minimal models with all-atom empirical forcefields. *Proteins* **75**, 430–41 (2009).

[13] Noel, J. K., Whitford, P. C. & Onuchic, J. N. The Shadow Map: A General Contact Definition for Capturing the Dynamics of Biomolecular Folding and Function. *J. Phys. Chem. B* **116**, 8692–8702 (2012).

[14] Noel, J. K., Whitford, P. C., Sanbonmatsu, K. Y. & Onuchic, J. N. SMOG@ctbp: simplified deployment of structure-based models in GROMACS. *Nuc. Acids Res.* **38**, W657–61 (2010).

[15] Culver, G. M., Cate, J. H., Yusupova, G. Z., Yusupov, M. M. & Noller, H. F. Identification of an RNA-protein bridge spanning the ribosomal subunit interface. *Science (New York, N.Y.)* **285**, 2133–2136 (1999).

[16] Noel, J. K., Chahine, J., Leite, V. B. P. & Whitford, P. C. Capturing Transition Paths and Transition States for Conformational Rearrangements in the Ribosome. *Biophys. J.* **107**, 2881–2890 (2014).

[17] Whitford, P. C. *et al.* Accommodation of aminoacyl-tRNA into the ribosome involves reversible excursions along multiple pathways. *RNA* **16**, 1196–1204 (2010).

[18] Whitford, P. C. & Sanbonmatsu, K. Y. Simulating movement of tRNA through the ribosome during hybrid-state formation. *J. Chem. Phys.* **139**, 121919 (2013).

[19] Mohan, S., Donohue, J. P. & Noller, H. F. Molecular mechanics of 30S subunit head rotation. *Proc. Natl. Acad. Sci. USA* **111**, 13325–30 (2014).

[20] Budkevich, T. V. *et al.* Regulation of the mammalian elongation cycle by subunit rolling: a eukaryotic-specific ribosome rearrangement. *Cell* **158**, 121–31 (2014).
